# Cardiovascular Aggression by Doxorubicin: The Search for Mechanisms

**DOI:** 10.5935/abc.20180214

**Published:** 2018-11

**Authors:** Wolney de Andrade Martins

**Affiliations:** Universidade Federal Fluminense, Niterói, RJ - Brazil

**Keywords:** Cardiovascular Diseases/complications, Neoplasms, Cardiotoxicity, Doxorubicin, Cyclophosphamida, Drug-Related Side Effects and Adverse Reactions, Vascular Stiffness, Ventricular Dysfunction

Cardio-oncology is an emerging subject in cardiology events and journals. Increased cancer (CA) incidence and survival rates, easier access to health care and the multiplicity of chemotherapy regimens all contribute to the increase in the diagnosis of cardiovascular complications in patients with CA. The increase in CA prevalence and mortality, as well as its cardiovascular complications, is the price that nations pay for the aging of their populations in an oncogenic environment.^1^ Therefore, we are faced with an epidemiological problem and major clinical challenges.

The discovery, in the 1960s, in the Adriatic Sea coast, of a red pigment produced by a fungus with great cytotoxic power has changed paradigms and introduced the concept of cure in clinical cancerology.^2^ Reports of toxicity previously presented with other chemotherapeutic agents had also been confirmed with the new class of anthracyclines. The novelty was the real possibility of cure. In the risk-benefit evaluation, adverse effects were neglected on behalf of the decision to use it.^3^ Warnings on the cardiotoxicity of doxorubicin (DOXO) came with the description of the classic ‘von Hoff curve’, where the risk of heart failure incidence at cumulative doses above 500 mg/m^2^ was demonstrated.^4^

Initially, the mechanism was said to be an oxidative effect of the chemotherapeutic agent. Later, it was demonstrated that DOXO had a blocking effect on topoisomerases II alpha (neoplastic cells) and beta (cardiomyocyte), as well as its consequence to the structure of DNA, which caused cell death.^5^ Other mechanisms of cellular aggression did not become clear until recently, when the action on the mechanical properties of cancerous and healthy cells was demonstrated, especially their effect on the cellular membrane.^6^

Today, there is a pertinent criticism about the lack of studies using judicious methodology and satisfactory casuistry in cardio-oncology. Indeed, there is a lack of basic science research addressing the aggression mechanisms in cardiovascular disease in CA patients. The study published in Arq Bras Cardiol^7^ investigates the relationships between arterial stiffness and ventricular dysfunction in patients undergoing DOXO and cyclophosphamide.

Theoretically, the cytotoxic impairment of DOXO could affect the endothelium, with consequences to blood pressure variables, secondarily becoming one of the multiple ventricular myocardial aggressions. Actually, there are no significant clinical reports of arterial hypertension in DOXO users, unlike patients undergoing angiogenesis inhibitors that act by blocking one of several endothelial growth pathways.^8^^,^^9^ The decision to study DOXO is justified by the high prevalence of its use in solid tumors such as breast cancer and hematological tumors.

In the cohort studied, which comprised 24 middle-aged women, high global cardiovascular risk was clearly observed. On average, the women were hypertensive and obese. There was no change in blood pressure variables in left ventricular function according to measurements by pulse wave velocity and two-dimensional echocardiography. The negative result should not be viewed with dismay. We need all the information we can find on these mechanisms. It is urgent to understand the pathophysiology of these cardiovascular aggressions. Only then will we be able to design ethical clinical trials with the highest possibility of results that might interfere with the reduction of cardiovascular lesions and, above all, with the improvement of survival of patients with cancer.
